# Behandlungen des Blasenschmerzsyndroms: eine Netzwerkmetaanalyse

**DOI:** 10.1007/s00120-021-01588-4

**Published:** 2021-07-05

**Authors:** Sebastian Graf

**Affiliations:** 1 Kepler Universitätsklinikum GmbH, Med Campus II, Krankenhausstraße 7a, 4020 Linz, Österreich; 2 UroEvidence@DGU, Berlin, Deutschland

## Originalpublikation

Imamura M, Scott NW, Wallace SA, Ogah JA, Ford AA, Dubos YA, Brazzelli M. Interventions for treating people with symptoms of bladder pain syndrome: a network meta-analysis. Cochrane Database of Systematic Reviews 2020, Issue 7. Art. No.: CD013325. 10.1002/14651858.CD013325.pub2.

## Hintergrund.

Das Blasenschmerzsyndrom („bladder pain syndrome“, BPS), das auch die interstitielle Zystitis (IC) umfasst, ist ein bislang unzureichend verstandenes klinisches Beschwerdebild, im Rahmen dessen sich Patienten mit verschiedenen Symptomen präsentieren. Die Behandlung des BPS ist sowohl für Patienten als auch für Ärzte herausfordernd. Zurzeit gibt es keine allgemein anerkannte Diagnose und es werden verschiedene Ursachen diskutiert. Dies spiegelt sich wider in einer Breite an Behandlungsoptionen, die (einzeln oder in Kombination und mit begrenzter Evidenz) eingesetzt werden. Eine Netzwerkmetaanalyse (NMA), mit der mehrere Behandlungen gleichzeitig verglichen werden, könnte dazu beitragen, die besten Therapieoptionen für Patienten mit BPS zu ermitteln.

## Ziele.

Ziel dieses Reviews war die Durchführung einer NMA zur Begutachtung der Effekte verschiedener Interventionen zur Behandlung von Patienten mit BPS.

## Methoden der Suche.

Wir durchsuchten am 11. Mai 2018 das Cochrane Incontinence Specialised Register, das Studien aus dem Cochrane Central Register of Controlled Trials (CENTRAL, in der Cochrane Library), MEDLINE, MEDLINE In-Process, MEDLINE Epub Ahead of Print, ClinicalTrials.gov sowie der World Health Organization International Clinical Trials Registry Platform (WHO ICTRP) enthält, und führten eine Handsuche in ausgewählten Journals, von Kongressbeiträgen und den Referenzlisten relevanter Fachartikel durch. Eine weitere Suche wurde am 5. Juni 2019 durchgeführt. Diese ergab vier kleine Studien, die für den Einschluss begutachtet, jedoch nicht in den Review eingeschlossen wurden.

## Auswahlkriterien.

Die Reviewautoren schlossen randomisierte kontrollierte Studien (RCT) und quasirandomisierte kontrollierte Studien (Quasi-RCT) zu Interventionen zur Behandlung von Erwachsenen mit BPS ein. Für den Einschluss wurden alle Arten von Interventionen (konservative, pharmakologische und chirurgische) berücksichtigt.

## Datenerhebung und Analyse.

Das Risiko für Bias bei eingeschlossenen Studien wurde mit dem Cochrane „Risk of bias“-Instrument bewertet. Die primären Endpunkte waren die Anzahl an Patienten, die eine Heilung oder Linderung erfuhren, sowie Schmerzen, die Harndrangfrequenz sowie nächtlicher Harndrang. Für jeden Endpunkt wurden Random-effects-NMA-Modelle mittels der Software WinBUGS 1.4 berechnet. Die Reviewautoren betrachteten die medianen Chancenverhältnisse (Odds Ratio; OR) für binäre Endpunkte sowie die Mittelwertdifferenzen („mean differences“, MD) für kontinuierliche Endpunkte, mit den dazugehörigen 95%igen Glaubwürdigkeitsintervallen („credible intervals“, CrIs). Die Ergebnisse der NMA wurden der direkten Evidenz aus paarweisen Metaanalysen von Studien zu direkten Vergleichen gegenübergestellt. Die Vertrauenswürdigkeit der Evidenz in den ausgewählten Behandlungskategorien wurde mit dem CINeMA-Tool bewertet.

## Ergebnisse.

Die Reviewautoren schlossen 81 RCT mit insgesamt 4674 Teilnehmern ein, wobei die mittlere Teilnehmerzahl bei 38 Teilnehmern (Range 10–369) je RCT lag. Die meisten Studien verglichen die Intervention mit einer Kontrollgruppe, nur wenige verglichen zwei aktive Behandlungen. Es gab 65 unterschiedliche Behandlungen, und die Ergebnisse einiger Vergleiche entstammten der direkten Evidenz aus nur einer Studie. Zur Vereinfachung wurden die Behandlungen nach ihrer Wirkweise in 31 Kategorien eingeteilt. Ein Großteil der Studien wies in den meisten Biasdomänen ein unklares oder hohes Risiko für Bias auf, insbesondere für Selektions- oder Detektionsbias. Zusammengefasst erbrachte die NMA Hinweise darauf, dass sechs (bezogen auf den Anteil an Heilung/Verbesserung der Beschwerden), eine (Schmerz), eine (Häufigkeit des Harndrangs) sowie keine (nächtlicher Harndrang) Behandlungskategorien im Vergleich zu den Kontrollgruppen wirksam waren, es bestand jedoch eine große Unsicherheit bezogen auf die Effektschätzungen.

Aufgrund der hohen Anzahl an Interventionsvergleichen in diesem Review konzentrierten sich die Autoren auf drei Interventionen: Antidepressiva (AD), Pentosanpolysulfat (PPS) sowie die neuromuskuläre Blockade. Diese Auswahl ergab sich aus der starken Empfehlung für diese Verfahren in den EAU-Leitlinien zur Behandlung des Blasenschmerzsyndroms [1].

Die Reviewautoren fanden Evidenz von sehr niedriger Vertrauenswürdigkeit dafür, dass Antidepressiva im Vergleich zu einer Kontrollgruppe möglicherweise mit einer größeren Wahrscheinlichkeit für eine Heilung oder Besserung der Beschwerden assoziiert sind (OR 5,91; 95 %-CrI 1,12 bis 37,56; sehr niedrige Vertrauenswürdigkeit der Evidenz), jedoch war unklar, ob AD zu einer Verringerung von Schmerzen (MD −1,27; 95 %-CrI −3,25 bis 0,71; niedrige Vertrauenswürdigkeit der Evidenz), der Harndrangfrequenz (MD −2,41; 95 %-CrI −6,85 bis 2,05; sehr niedrige Vertrauenswürdigkeit der Evidenz) oder des nächtlichen Harndrangs (MD 0,01; 95 %-CrI −2,53 bis 2,50; sehr niedrige Vertrauenswürdigkeit der Evidenz) führten.

Es gab keine Evidenz dafür, dass PPS zur Heilung oder Linderung der Beschwerden (OR 0,14; 95 %-CrI 0,40 bis 3,35; sehr niedrige Vertrauenswürdigkeit der Evidenz), weniger Schmerzen (MD 0,42; 95 %-CrI −1,04 bis 1,91; niedrige Vertrauenswürdigkeit der Evidenz), weniger häufigem Harndrang (MD −0,37; 95 %-CrI −5,00 bis 3,44; sehr niedrige Vertrauenswürdigkeit der Evidenz) oder weniger nächtlichem Harndrang (MD −1,20; 95 %-CrI −3,62 bis 1,28; sehr niedrige Vertrauenswürdigkeit der Evidenz) führte.

Es gab Evidenz dafür, dass eine neuromuskuläre Blockade häufiger zu Heilung oder Linderung der Beschwerden (OR 5,80; 95 %-CrI 2,08 bis 18,30) führte, nicht jedoch zu einer Verringerung von Schmerzen (MD −0,33; 95 %-CrI −1,71 bis 1,03), der Harndrang-Frequenz (MD −0,91; 95 %-CrI −3,24, 1,29) oder des nächtlichen Harndrangs (MD −0,04; 95 %-CrI −1,35 bis 1,27). Die Vertrauenswürdigkeit dieser Evidenz wurde durchgängig als sehr niedrig bewertet.

## Schlussfolgerungen der Autoren.

Für die Reviewautoren konnte die Wirksamkeit einiger Behandlungen für Patienten mit BPS nicht sicher dargestellt werden, da die Vertrauenswürdigkeit der Evidenz generell niedrig bis sehr niedrig war. Da die Anzahl an Studien relativ hoch war, waren viele Daten verfügbar, die extrahiert werden konnten. Allerdings waren die Stichprobengrößen bei vielen Studien klein, sodass die Wirksamkeit der Behandlungen oftmals nicht präzise beurteilt werden konnte (ungenaue Effektschätzungen). Die NMA konnte erfolgreich durchgeführt werden, jedoch beeinträchtigte die kleine Anzahl an Studien in jeder Behandlungskategorie die Möglichkeit, alle Vorteile dieser Analyseform auszuschöpfen. Größere, fokussiertere Studien sind erforderlich, um die aktuelle Evidenzbasis zu verbessern.

## Kommentar

Das BPS ist ein chronisches Zustandsbild, welches am häufigsten Frauen betrifft. Es ist charakterisiert durch Schmerzen im Bereich der Harnblase oder des kleinen Beckens sowie weitere Beschwerden des Harntrakts, wie häufigen und/oder nächtlichen Harndrang [2]. Die Ursachen des BPS bleiben weitgehend ungeklärt. Bisher konnte kein einzelner Auslöser oder validierter diagnostischer Parameter des Zustandbildes identifiziert werden. Daher basiert die Diagnose meist auf anamnestischen Erhebungen oder ergibt sich durch Ausschluss anderer identifizierbarer Ursachen [2]. Darüber hinaus gibt es Hinweise, dass das BPS histopathologisch subtypisiert werden kann in zwei Gruppen, bei welchen auch Experimente im Tiermodell unterschiedliche zugrunde liegende Pathomechanismen vermuten lassen. Einerseits solche, in denen Hunner-Läsionen festgestellt werden können, wo sich charakteristische histopathologische Änderungen im Urothel mit epithelialer Denudation, verstärkter lokaler Immunantwort und Infiltration von B‑Zellen finden und dadurch auf eine lokale Dysfunktion des Urothels schließen lassen. Andererseits eine solche Gruppe ohne zystoskopische Auffälligkeiten, bei welcher die lokal-inflammatorische Komponente nicht im Vordergrund steht, sondern möglicherweise neurophysiologische Ursachen oder somatoforme Transformationen führend sind [3]. Möglicherweise handelt es sich dabei tatsächlich um verschiedene Krankheiten.

In der Vergangenheit wurden die diagnostischen Kriterien für BPS und IC mehrfach überarbeitet. 1987 wurde die IC als Ergebnis objektiver Parameter in der Zystoskopie und Urodynamik definiert [4]. Dieser diagnostische Weg stellte sich in der Praxis als zu umständlich und risikoreich heraus [5], sodass die internationale Kontinenzgesellschaft (ICS) 2002 eine breitere Definition des „schmerzhaften Blasensyndroms“ vorschlug [6, 7]. Diese Definition resultierte im heute gängigen Terminus des BPS, welcher von der ESSIC geprägt wurde. Er definiert sich als „chronischer Beckenschmerz, Druckgefühl oder Unbehagen über einen Zeitraum von mehr als 6 Monaten, wahrgenommen in der Harnblase und begleitet von mindestens einem anderen Miktionssymptom wie anhaltendem Harndrang oder häufigem Harnlassen“ [8].

Durch diese bis zuletzt verschiedenen Definitionen sind genaue epidemiologische Aussagen schwierig. Einer Schätzung nach beläuft sich die Prävalenz bei Frauen auf 100–200 pro 100.000 und bei Männern auf etwa ein Zehntel dieses Werts [2].

Eine große Anzahl verschiedener therapeutischer Ansätze wird in der Behandlung des BPS verfolgt [2]. Initial werden meist neben Patientenedukation und Lifestyle-Modifikation diätetische Maßnahmen, Physiotherapie und Analgetika eingesetzt. In therapierefraktären Stadien wird dies ergänzt durch pharmakologische Therapien wie Analgetika höherer Potenz, Antidepressiva, Antibiotika und Immunmodulatoren. Chirurgische Techniken wie Botulinumtoxin-Injektion oder Entfernung der Harnblase stellen die letzten Therapielinien dar [2]. Aktuelle Leitlinien empfehlen prinzipiell einen patientenbasierten, individualisierten Zugang [9].

Diese Breite an verschiedenen Behandlungen macht eine NMA zu einem geeigneten Instrument, gleichzeitig die Evidenzlage dieser Strategien darzustellen und zu vergleichen. Die vorliegende Übersichtsarbeit stellt dies dar.

### Evidenzanalyse.

Die hier diskutierte Cochrane-Übersichtsarbeit wurde in Form einer NMA veröffentlicht. Die Literaturrecherche erfolgte in anfangs genannten Datenbanken, ohne Einschränkungen hinsichtlich der Sprache. Auch Ahead-of-print-Artikel, welche auf MEDLINE gelistet waren, wurden inkludiert. Insgesamt identifizierten die Autoren nach Duplikatkorrektur und Berücksichtigung der Ausschlusskriterien 431 Arbeiten, von welchen letztendlich 81 in den Review und 57 in die NMA eingeflossen sind. Die Auswahl der Studien erfolgte von zwei Autoren unabhängig voneinander im Vier-Augen-Prinzip durch Sichtung von Titeln/Abstracts und danach der Volltexte. Die Qualitätsbewertung der einzelnen Studien erfolgte nach den üblichen Standards der Cochrane Collaboration. Dabei wurde die Evidenzqualität im besten Fall als moderat, meist jedoch als niedrig bis sehr niedrig bewertet. Hauptsächlich dafür verantwortlich waren laut den Autoren das hohe Risiko für Verzerrung („bias“ – hier v. a. aufgrund von „performance bias“, „detection bias“ und „attrition bias“) sowie Ungenauigkeiten bei den Effektschätzern und Heterogenität zwischen den Studien.

### Wichtigste Ergebnisse.

Die meisten therapeutischen Interventionen beeinflussten die subjektive Wahrnehmung der Beschwerdeintensität, nur wenige konnten eine Änderung in objektiven Parametern wie Schmerz oder Häufigkeit des Harndrangs darstellen. Im Rahmen der eingeschränkten Aussagequalität der Ergebnisse dieses Reviews konnten Hinweise darauf gefunden werden, dass Antidepressiva sowie PPS möglicherweise mit einer größeren Wahrscheinlichkeit auf Heilung oder Besserung der Beschwerden assoziiert sind, verglichen mit Kontrollgruppen. Mit einer moderaten Aussagequalität konnte dargestellt werden, dass Schmerz am ehesten von physikalischer Therapie positiv beeinflusst wurde.

### Limitationen.

Die wichtigsten Limitationen der diskutierten Übersichtsarbeit ergeben sich aus der eingeschränkten Qualität der einzelnen eingeschlossenen Studien. Diese ergaben sich hauptsächlich aus kurzen Nachbeobachtungszeiträumen, so erfolgte bei lediglich sechs Studien ein Follow-up von 12 Monaten oder mehr. Ein weiterer wichtiger Faktor waren kleine Studienpopulationen: In nur fünf der eingeschlossenen Studien wurden mehr als 100 Teilnehmer untersucht, der Median belief sich auf 38 Teilnehmer. In Verbindung mit den heterogenen Endpunkten lassen sich daraus nur Aussagen mit sehr eingeschränkter Sicherheit ableiten. Durch die Heterogenität in der Art, wie in den einzelnen Studien über unerwünschte Ereignisse berichtet wurde, konnte darüber keine Metaanalyse durchgeführt werden. Daher besteht Unsicherheit bezüglich des Auftretens von unerwünschten Ereignissen.

### Ausblick.

Die eingeschränkte Aussagekraft der vorliegenden NMA resultiert nicht aus methodischen Schwächen, sondern aus der heterogenen Studienlandschaft dieses uneinheitlich definierten Zustandsbilds. Hier sind adäquat große, qualitativ hochwertige Studien mit engerer Zielsetzung notwendig. Um eine reproduzierbare, wirksame Therapie des BPS zu finden, ist es jedoch zunächst vorrangig, die Pathophysiologie dieses komplexen Zustandsbilds besser zu verstehen, sowie die Interrelation mit anderen chronischen Beckenschmerzsyndromen. Eine bessere klinische Phänotypisierbarkeit und Abgrenzbarkeit, notwendigerweise durch objektive diagnostische Parameter, ist nötig, um die verfügbaren Therapiewege auf den einzelnen Patienten abzustimmen.

## Fazit für die Praxis.

- Es besteht eine große Heterogenität in den Behandlungsformen des BPS („bladder pain syndrome“).
- Möglicherweise führen Behandlungen mit Antidepressiva und Pentosanpolysulfat mit einer größeren Wahrscheinlichkeit zu einer Linderung oder zur Heilung von Beschwerden bei Patienten mit BPS.
- Es besteht Unsicherheit bezüglich des Auftretens von unerwünschten Ereignissen während den untersuchten Interventionen.
- Die Sicherheit der Aussagen zu den einzelnen Endpunkten in dieser Übersichtsarbeit wurde aufgrund eingeschränkter Evidenzqualität mehrheitlich als gering bis sehr gering eingeschätzt.
- Grundlegende Fragen zur Ätiologie und Pathophysiologie des BPS sind noch zu klären, bevor mit reproduzierbaren Therapieerfolgen zu rechnen ist.

## Abkürzungen

AD: Antidepressiva
BPS: Blasenschmerzsyndrom, „bladder pain syndrome“
CrI: Glaubwürdigkeitsintervall, „credible interval“
EAU: European Association of Urology
ESSIC: European Society for the Study of Interstitial Cystitis (heute: International Society for the Study of Bladder Pain Syndrome)
IC: Interstitielle Zystitis, „interstitial cystitis“
MD: Mittlerer Unterschied, „mean difference“
OR: Chancenverhältnis, Odds Ratio
PPS: Pentosanpolysulfat
